# miR‐15a‐5p, miR‐15b‐5p, and miR‐16‐5p inhibit tumor progression by directly targeting MYCN in neuroblastoma

**DOI:** 10.1002/1878-0261.12588

**Published:** 2019-11-29

**Authors:** Srinivas Chava, C. Patrick Reynolds, Anup S. Pathania, Santhi Gorantla, Larisa Y. Poluektova, Don W. Coulter, Subash C. Gupta, Manoj K. Pandey, Kishore B. Challagundla

**Affiliations:** ^1^ Department of Biochemistry and Molecular Biology & the Fred and Pamela Buffett Cancer Center University of Nebraska Medical Center Omaha NE USA; ^2^ Childhood Cancer Repository Texas Tech University Health Sciences Center Lubbock TX USA; ^3^ Department of Pharmacology and Experimental Neuroscience University of Nebraska Medical Center Omaha NE USA; ^4^ Department of Pediatrics Division of Hematology/Oncology University of Nebraska Medical Center Omaha NE USA; ^5^ Department of Biochemistry Institute of Science Banaras Hindu University Uttar Pradesh India; ^6^ Department of Biomedical Sciences Cooper Medical School of Rowan University Camden NJ USA

**Keywords:** Ago2, microRNA, MYCN, neuroblastoma, patient‐derived xenografts

## Abstract

Neuroblastoma (NB) is the most common extracranial solid malignancy in children. Despite current aggressive treatment regimens, the prognosis for high‐risk NB patients remains poor, with the survival of less than 40%. Amplification/stabilization of MYCN oncogene, in NB is associated with a high risk of recurrence. Thus, there is an urgent need for novel therapeutics. The deregulated expression of microRNA (miR) is reported in NB; nonetheless, its effect on *MYCN* regulation is poorly understood. First, we identified that miR‐15a‐5p, miR‐15b‐5p, and miR‐16‐5p (hereafter miR‐15a, miR‐15b or miR‐16) were down‐regulated in patient‐derived xenografts (PDX) with high *MYCN* expression. MiR targeting sequences on MYCN mRNA were predicted using online databases such as TargetScan and miR database. The R2 database, containing 105 NB patients, showed an inverse correlation between MYCN mRNA and deleted in lymphocytic leukemia (*DLEU*) 2, a host gene of miR‐15. Moreover, overexpression of miR‐15a, miR‐15b or miR‐16 significantly reduced the levels of MYCN mRNA and N‐Myc protein. Conversely, inhibiting miR dramatically enhanced MYCN mRNA and N‐Myc protein levels, as well as increasing mRNA half‐life in NB cells. By performing immunoprecipitation assays of argonaute‐2 (Ago2), a core component of the RNA‐induced silencing complex, we showed that miR‐15a, miR‐15b and miR‐16 interact with MYCN mRNA. Luciferase reporter assays showed that miR‐15a, miR‐15b and miR‐16 bind with 3’UTR of MYCN mRNA, resulting in *MYCN* suppression. Moreover, induced expression of miR‐15a, miR‐15b and miR‐16 significantly reduced the proliferation, migration, and invasion of NB cells. Finally, transplanting miR‐15a‐, miR‐15b‐ and miR‐16‐expressing NB cells into NSG mice repressed tumor formation and *MYCN* expression. These data suggest that miR‐15a, miR‐15b and miR‐16 exert a tumor‐suppressive function in NB by targeting *MYCN*. Therefore, these miRs could be considered as potential targets for NB treatment.

## Graphical abstract

A model summarizing how miR‐15a, miR‐15b, and miR‐16 suppress tumor progression in neuroblastoma by targeting MYCN. When miRs are overexpressed, argonaute‐2‐mediated interaction of miR with MYCN mRNA increases, followed by degradation of MYCN, leading to neuroblastoma regression.

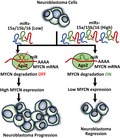

**Abbreviations**
Act‐D: actinomycin D
Ago2: argonaute2
COG: children’s oncology group
Ctrl: control
DKK3: dickkopf‐related protein 3
DLEU2: deleted in lymphocytic leukemia 2
IACUC: institutional animal care and use committee
IP: immunoprecipitation
LNA: locked nucleic acid
miR: microRNA
MTT: 3‐(4,5‐dimethylthiazol‐2‐yl)‐2,5‐diphenyl tetrazolium bromide
MYCN: v‐myc myelocytomatosis viral related oncogene, neuroblastoma derived (avian)
NB: neuroblastoma
NSG: NOD.Cg‐PrkdcscidIl2rgtm1Wjl/SzJ
P: passage
PDX: patient‐derived xenografts
qRT‐PCR: quantitative reverse transcription PCR
RISC: RNA‐induced silencing complex
Scr: scrambled
snoRNA: small nucleolar RNA

## 1 Introduction

Neuroblastoma (neuro = nerve; blastoma = cancer; NB) is a rare type of tumor that develops in neuroblasts located in the adrenal glands (Cushing and Wolbach, 1927). NB is one of the most common childhood cancers, and is unfortunately associated with approximate 10% of all pediatric cancer deaths (Cushing and Wolbach, 1927; Johnsen *et al.*, 2019; Nakagawara *et al.*, 2018; Whittle *et al.*, 2017). Every year, 800 new cases of NB are diagnosed. Generally, infants and young children, but rarely children older than ten years, develop NB, and it is more common in boys. The NB primary tumor often occurs in the abdomen, and a number of clinical and biological factors are used to define risk categories (Amati *et al.*, 1993; Blackwood and Eisenman, 1991; Dang, 2012). Standard treatment for high‐risk NB includes the combination of chemotherapy, surgical resection, autologous stem cell transplantation, radiation therapy, and immunotherapy (Bagatell and Cohn, 2016; Whittle *et al.*, 2017). However, high‐risk/aggressive NB patients often develop resistance to chemotherapeutic drugs due to the interaction of NB cells with the tumor microenvironment (Challagundla *et al.*, 2015). The overall 5‐year survival rate in children with high‐risk disease is < 40%, representing a cause of the majority of the cancer‐related deaths in infants (Berthold *et al.*, 2017; Coughlan *et al.*, 2017; Di Cataldo *et al.*, 2017; Moreno *et al.*, 2016).

The molecular hallmark of high‐risk NB is the aberrant expression of the proto‐oncogene *MYCN*, which is located on the short arm of chromosome 2p24 (Schwab *et al.*, 1983). *MYCN* belongs to the proto‐oncogenes MYC family, which includes *MYC, MYCL*, and *MYCN* and translates c‐Myc, L‐Myc, and N‐Myc proteins, respectively. The aberrant expression of MYC family members are critical in the pathogenesis of a variety of malignancies including small cell lung cancer, glioblastoma, retinoblastoma, medulloblastoma, and prostate cancer (Beltran *et al.*, 2011; Brodeur *et al.*, 1984; Cotterman *et al.*, 2008; Kim *et al.*, 2006; Nesbit *et al.*, 1999; Yoshida, 2018; Zlotorynski, 2018). Studies have demonstrated that high expression/amplification of *MYCN* is associated with increased energy metabolism, rapid tumor growth, short survival rates, unfavorable histology, and chemotherapy resistance in NB patients (Chan *et al.*, 1997; Huang and Weiss, 2013; Wenzel *et al.*, 1991). Yet, no small molecule inhibitors are available to target *MYCN* in the clinic (Chen *et al.*, 2008).

MicroRNA (miR) are small noncoding RNA molecules that regulate gene expressions by promoting mRNA degradation and/or repression of translation. The miR expression is dysregulated in the majority of human cancers, including NB (Bray *et al.*, 2009; Challagundla *et al.*, 2015). Ectopic expression of *MYCN*, as well as primary tumors with *MYCN* amplification, express an up‐regulation of a specific miR signature that correlates with a poor prognosis and may positively contribute to NB pathogenesis (Mestdagh *et al.*, 2010; Schulte *et al.*, 2008). Furthermore, *MYCN* inhibits tumor suppressor p21 levels by up‐regulation of the miR‐17‐5p‐92 cluster members and positively correlates with poor patient survival in NB. This portrays the activation of *MYCN* and miR pathways (Bray *et al.*, 2009; Mestdagh *et al.*, 2010). Treatment with a differentiating agent, 13‐cis‐retinoic acid, induces a down‐regulation of *MYCN* and up‐regulation of a specific miR set in NB cells (Chen and Stallings, 2007). Recent studies have shown that *MYCN*‐amplified NB tumors express lower levels of miR‐29a that directly target an immune checkpoint member B7‐H3 (CD276) and facilitate neoplastic neuroblast cells to escape the immune surveillance (Espinosa‐Parrilla *et al.*, 2014; Xu *et al.*, 2009). The miR‐15a/16‐1 cluster is located in the intronic region of *deleted in leukemia* (*DLEU*) *2* gene (Klein *et al.*, 2010). These are expressed in a wide variety of cells and are evolutionarily conserved. Deleted in lymphocytic leukemia 2 (DLEU2) is located on chromosome 13 at 13q14.2. The loss of the miR‐15a/16‐1 locus has been shown to cause human chronic lymphocytic leukemia and acute myeloid leukemia in a mouse model (Klein *et al.*, 2010; Lovat *et al.*, 2018). A transcription factor B‐cell‐specific activator protein inhibits the expression of *miRNA‐15a/16‐1* by direct interaction with *Dleu2* (Kasar *et al.*, 2014).

Therefore, it is crucial to understand the regulation of *MYCN* by miR in high‐risk NB. Here we investigated the specific miR involved in the regulation of *MYCN* expression, and their mechanism of action, differential expression, and effects on the functional properties of the NB cells using *in vitro* and *in vivo *models. We employed NB‐specific patient‐derived xenografts (PDX), cell line‐based xenografts, and NB cells.

## 2 Materials and methods

### 2.1 Expansion of NB PDX tumors

Neuroblastoma‐specific PDX tumors, namely, children’s oncology group (COG)‐N‐415x, COG‐N‐440x, COG‐N‐561x, COG‐N‐660x, COG‐N‐620x, and COG‐N‐426x, were established in the laboratory of our collaborator Dr. C. Patrick Reynolds. Deidentified tumor tissues were received through the Children's Oncology Group Cell Culture and Xenograft Repository (http://www.cogcell.org). In brief, patient tumor tissues were surgically removed and cut into multiple pieces in sterile Hanks’ balanced salt solution supplemented with antibiotics. Tumor tissues were subcutaneously injected into 4‐ to 6‐week‐old Nude mice [passage (p) 0]. After xenograft transplantation, animals were observed for palpable tumors. When tumors reached ~ 1500 mm^3^, the animals were euthanized, and tumors were excised and passaged to another cohort of animals. The average time for tumor growth was 60 days (ranging from 30 to 120 days). Each tumor tissue was passaged into P0–P4 *in vivo* generations. At P4, tumor tissues were excised and used for RNA isolation. The study methodologies conformed to the standards set by the Declaration of Helsinki. The study methodologies were approved by the local ethics committee.

### 2.2 NB patient survival data analysis

R2, a web‐based genomics analysis, and visualization application platform (http://r2.amc.nl) developed by the Academic Medical Center in Amsterdam (The Netherlands) were used to investigate the expression of *MYCN, DLEU2* (miRNA‐15 host gene) and their relationship with overall survival probability. We obtained microarray analysis results from publicly available NB patient cohort data (Molenaar *et al.*, 2012)**.** Characteristics of the patients were published earlier (Molenaar *et al.*, 2012). Kaplan–Meier curves showing the effect of *MYCN* and *DLEU2* gene expression levels on survival probability such as higher or lower expression predicts poor overall survival probability were drawn using the R2 scan method. The relationship between *MYCN* and *DLEU2* was drawn using the R2 scan method and plotted.

### 2.3 NB cell lines and culture conditions

SK‐N‐BE(2), NB‐19, and SH‐EP Tet21N, doxycycline‐repressible (Tet‐Off) *MYCN* gene cells were cultured in Roswell Park Memorial Institute media containing 10% heat‐inactivated FBS (Sigma‐Aldrich, St. Louis, MO, USA). CHLA‐136 cells were cultured in Iscove's Modified Dulbecco's Medium containing 20% FBS and supplemented with 50 U of penicillin per mL, 0.1 mg of streptomycin per mL, l‐glutamine, sodium pyruvate, and non‐essential amino acids as previously described at 37 °C in a 5% CO_2_ humidified atmosphere. All cell lines were authenticated by DNA profiling before use (Challagundla *et al.*, 2015).

### 2.4 MiRNA reagents and transfections

Pre‐miR, pre‐miR‐scrambled (Scr), locked nucleic acid (LNA) anti‐miR, and LNA‐anti‐miR‐Scr oligonucleotides were obtained from Applied Biosystems (Foster City, CA, USA). MiR mimics or miR inhibitors were transfected into NB cells using silent Fect lipid reagent (Bio‐Rad Laboratories, Inc., Hercules, CA, USA) following the manufacturer's protocol. Cells were analyzed 48 h after transfection unless stated otherwise. MiRNA transfection efficiencies or the effect of the miR inhibitors were assayed by quantitative reverse transcription PCR (qRT‐PCR).

### 2.5 Measurement of MYCN mRNA stability

Neuroblastoma cells were transfected with anti‐Scr or anti‐miR for 24 h and treated with 2 μm actinomycin D (Act‐D) as the transcription inhibitor for the indicated time points. The cells were harvested and analyzed for relative levels of MYCN mRNA (normalized to GAPDH mRNA) using qRT‐PCR.

### 2.6 RNA isolation and qRT‐PCR assay

Total RNA was isolated from the cells using the PureLink™ RNA Mini Kit (Life Technologies, Carlsbad, CA, USA) according to the manufacturer’s instructions. Reverse transcription reactions were performed using iScript cDNA synthesis kit (Bio‐Rad) for mRNA, or TaqMan miR Reverse Transcription kit (Applied Biosystems) for mature miR as described earlier (Challagundla *et al.*, 2015). A qRT‐PCR assay was performed using an ABI StepOne real‐time PCR system (Applied Biosystems) with SYBR green mix (Bio‐Rad) for mRNA expression or TaqMan Universal Fast PCR master mix for miRNA, as described earlier (Challagundla *et al.*, 2011, 2015; Dai *et al.*, 2012; Sun *et al.*, 2012). All reactions were carried out in triplicate. Relative gene or miRNA expression levels were normalized with GAPDH mRNA or U6 small nucleolar RNA (snoU6) and were calculated using the ΔCτ method. The qRT‐PCR primer sequences for *MYCN* and glyceraldehyde‐3‐phosphate dehydrogenase (*GAPDH*) were as follows: (MYCN) Forward: 5′‐ACCCGGACGAAGATGACTTCT‐3′, Reverse: 5′‐CAGCTCGTTCTCAAGCAGCAT‐3′; (GAPDH) Forward: 5′‐GATTCCACCCATGGCAAATTC‐3′, Reverse: 5′‐AGCATCGCCCCACTTGATT‐3′ (Spandidos *et al.*, 2010; Wang and Seed, 2003).

### 2.7 Western blot analysis and antibodies

Rabbit monoclonal anti‐N‐Myc (D1V2A) and rabbit polyclonal anti‐GAPDH (G9) were obtained from Cell Signaling Technology and Santa Cruz Biotechnology. Mouse monoclonal anti‐argonaute‐2 (Ago2) (EPR10411) was purchased from Abcam, and mouse monoclonal anti‐Vinculin (V9131) was procured from Sigma‐Aldrich. The cell lysate preparation and immunoblotting were performed using the methods described before (Challagundla *et al.*, 2011, 2015).

### 2.8 *MYCN* 3’UTR constructs and luciferase reporter assays

Publicly available online bioinformatics databases such as TargetScan (http://www.targetscan.org), miRDB (http://mirdb.org/miRDB), and http://www.microrna.org were used to predict the potential miRNA binding sites in the 3′ UTR of MYCN mRNA. Predicted miR binding sites in the 3′ UTR region (909 bp) of MYCN mRNA (referred to as 3′ UTRwt) and mutations (seven bases) in the miRNA binding sites (seven bases) of the 3′ UTR region (referred to as 3′ UTR mut) were cloned in a luciferase vector pEZX‐MT06 (Cat. # HmiT117783‐MT06, Cat. # CS‐HmiT117783‐MT06‐01; GeneCopoeia, Rockville, MD, USA). An empty pEZX‐MT06‐luciferase vector was used as a negative control (Ctrl). NB cells were transfected with luciferase reporter plasmids with or without miR‐15a, miR‐15b, and miR‐16 oligonucleotides (25 nm), using PEI reagent. After 48 h, the cells were lysed and the luciferase activity was measured using the luciferase assay reagent (Promega, Madison, WI, USA) and the luminometer. The luciferase activity was expressed relative to negative Ctrl, which was set at 100 (Challagundla *et al.*, 2011, 2015).

### 2.9 Immunoprecipitation of Ago2 protein‐associated RNA (RNA IP)

Immunoprecipitation (IP) of Ago2‐bound RNA‐protein complexes were performed as previously described (Lopez de Silanes *et al.*, 2005; Tenenbaum *et al.*, 2002). In brief, NB cells were transfected with miR for 48 h and lysed in polysome buffer. The cleared supernatants were incubated with Ago2 primary antibody at 4 °C for 6 h, followed by incubation with protein A beads for an additional 2 h. After washing with NT2 buffer, the bead‐bound Ago2‐RNA was separated and used for the analysis of bound mRNA or miR as described (Challagundla *et al.*, 2011, 2015).

### 2.10 Cellular toxicity assay

The viability of NB cells transfected with miR mimics or Scr Ctrl mimics was examined using 3‐(4,5‐dimethylthiazol‐2‐yl)‐2,5‐diphenyl tetrazolium bromide (MTT)‐based cellular cytotoxicity assay kit (Promega) according to the manufacturer's instructions.

### 2.11 Wound healing assay

Cell migration was carried out *in vitro* using Ibidi wound healing assay kit according to the manufacturer’s instructions. NB cells were plated onto the Ibidi cell culture inserts and transfected with miR for 24 h. The inserts were taken out for fixation of cells. The images were captured under a microscope (Nikon, TS100; Nikon Corporation, Tokyo, Japan) at 0 and 24 h for the quantification of migrated cells in each group.

### 2.12 Transwell migration and invasion assay

To examine the effects of miR on cell invasion, we used a Matrigel invasion chamber and followed a method described by the manufacturer (BD Biosciences, San Jose, CA, USA). After transfection with miR for 48 h, NB cells were plated in the upper compartment of the Boyden chamber (8 μm) for 24 h. Plates were either coated with Matrigel (invasion assay) or not coated with Matrigel (migration assay). Migrated or invaded cells through the lower chamber were fixed with methanol. After staining with crystal violet, the migrated/invaded cells were imaged under a microscope. The migrated/invaded cells were counted from five different fields for each chamber and expressed as a percentage compared with the control.

### 2.13 *In vivo* xenograft experiments

All mouse experiments were carried out according to the protocols approved by the Institutional Animal Care and Usage Committee (IACUC) of UNMC. Six‐week‐old NOD.Cg‐PrkdcscidIl2rgtm1Wjl/SzJ (NSG) mice were obtained through Translational Mouse Model Core Facility (https://www.unmc.edu/vcr/cores/translational-cores/tmmcf/index.html) at UNMC. The mice were treated with 2 Gy total body irradiation for enhanced xenograft growth as previously described (Challagundla *et al.*, 2015). SK‐N‐B(E)2 cells stably expressing luciferase gene were transfected with miR‐15a, miR‐15b, miR‐16a or Scr Ctrl. The transfected cells were subcutaneously inoculated into the flank [4 × 10^6^ cells in 100 μL Matrigel™ (Becton Dickinson, Franklin Lakes, NJ, USA)]. All the mice were monitored daily to ensure that the injection sites were healthy and tumor volume was measured twice in a week. At day 45, the mice were sacrificed, and the tumors were excised, measured, and photographed. The final tumor volumes were determined using the equation: V = L × W^2^/2 (L: tumor length; W: tumor width). A portion of the excised tumor was used for RNA work and the other portion was used for western blotting.

### 2.14 Statistical analysis

The data were expressed as mean ± standard deviation (SD). The difference between the treatment groups was analyzed using Student's *t*‐test when only two groups were present, or was assessed by one‐way analysis of variance when more than two groups were compared. The data analysis was carried out using graph pad prism 7.0 software (Prism ‐ GraphPad, San Diego, CA, USA). A value of *P* < 0.05 was considered statistically significant.

## 3 Results

### 3.1 Higher *MYCN* is associated with lower miR‐15a, miR‐15b and miR‐16 in NB PDX tumors

**Figure 1:**
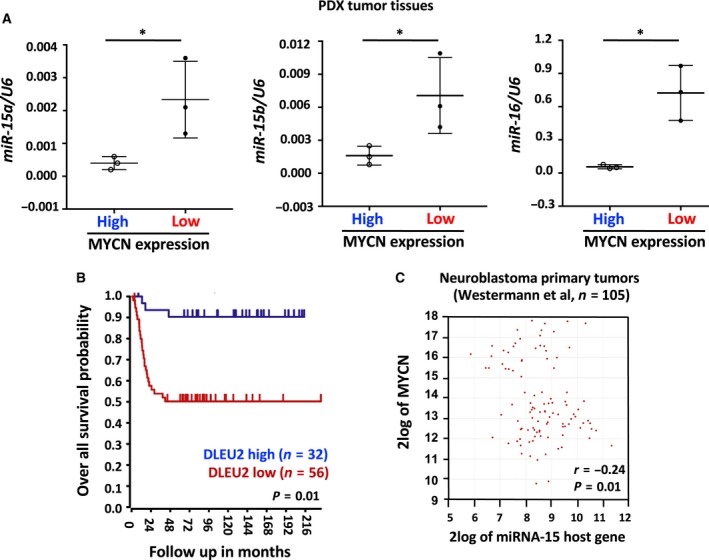
Higher *MYCN* associates with lower miR‐15a, miR‐15b, and miR‐16 levels in PDX tumor tissues. (A) A qRT‐PCR for miR‐15a, miR‐15b, and miR‐16 (normalized to snoRNA U6) in PDX tumors. (B) Kaplan–Meier curve showing the survival probability of NB patients (*n* = 88) with low *DLEU2*, a host gene to miR‐15. Patients with lower DLEU2 had a shorter survival. (C) Correlation between *DLEU2* and MYCN mRNA expression in primary tumors (*n* = 105). Data shown are means ± standard deviation from three independent biological experiments. *P < 0.05. The names of the statistical tests used to determine significance or lack thereof (if applicable) are given.

The differential expression of miR has been demonstrated in NB patients (Beckers *et al.*, 2015; Buechner and Einvik, 2012; Chen and Stallings, 2007; Galardi *et al.*, 2018; Gattolliat *et al.*, 2014; Haug *et al.*, 2015; Lin *et al.*, 2010; Megiorni *et al.*, 2017; Powers *et al.*, 2016; Schulte *et al.*, 2008; Shohet *et al.*, 2011). An extensive literature search revealed that the expression of tumor suppressor miR, particularly the miR‐15 family, has a potential connection to *MYCN* as regards prognosis, acquired resistance, and NB stages. Therefore, we focused on the miR‐15 family members miR‐15a, miR‐15b, and miR‐16. We have access to PDX tumors (*n* = 6) from the Childhood Cancer Repository through our collaborator Dr. Patrick Reynolds. At first, we expanded PDX tumors in nude mice as illustrated in Fig. S1A. We first tested the MYCN mRNA expression levels by qRT‐PCR in PDX tumors, dichotomized into high and low *MYCN* tumors, and then examined the levels of miR. We found that the levels of miR‐15a, miR‐15b, and miR‐16 in PDX tumors were inversely correlated with *MYCN* expression (Fig. 1A). Since these results are derived from six PDX tumors, future studies using additional tumors are required. 

Next, we investigated the effects of *MYCN* and miR on the survival probability of NB patients in an independent set of primary tumors (*n* = 88) using the R2 database (http://r2.amc.nl). For this, we examined the expression pattern of *MYCN* along with *DLEU2*, which is a host gene of miR‐15. Based on *MYCN* and *DLEU2* expression in NB patients, survival curves were plotted and survival probability was estimated (Tumor NB public‐Versteeg‐88‐MAS5.0‐u133p2). Survival curves for DLEU and MYCN are depicted in Figs 1B and S1B, respectively. We found that lower *DLEU2* (Fig. 1B) and higher *MYCN* (Fig. S1B) expression were associated with poor patient survival. Because *DLEU2* is the host gene of miR‐15, it can be concluded that miR‐15 positively correlates with NB patient survival. 

Further, we investigated the relationship between the expression of *MYCN* and *DLEU2* in an independent set of NB primary tumors (Tumor NB public‐Westermann‐105‐ag44kcwolf) using the R2 database. We noted that higher *MYCN* was associated with lower *DLEU2*, suggesting an inverse correlation between the two in NB patients (*n* = 105, *r* = −0.24, *P = *0.01; Fig. 1C).

### 3.2 MiR‐15a, miR‐15b, and miR‐16 regulate *MYCN*

**Figure 2:**
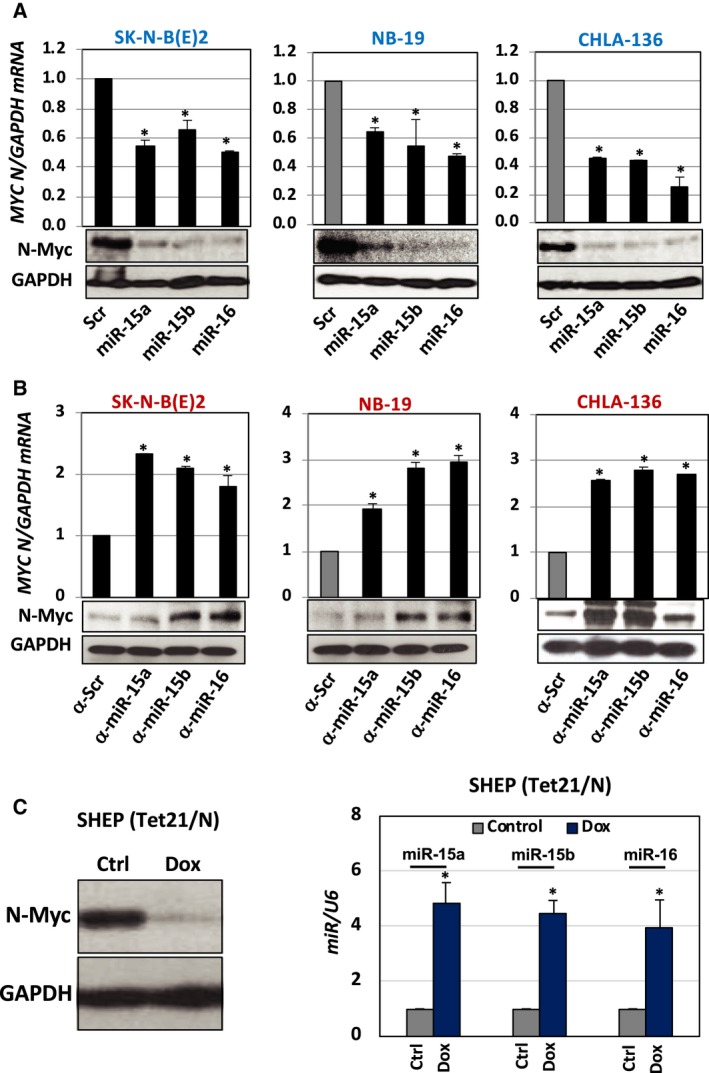
MiR‐15a, miR‐15b, and miR‐16 regulate MYCN in NB cells. (A,B) A qRT‐PCR for MYCN mRNA and western blotting for N‐Myc protein in NB cells [SK‐N‐BE(2), NB‐19 and CHLA‐136] transfected with either (A) miR‐15a, miR‐15b or miR‐16 oligonucleotides (Scr as Ctrl) or (B) inhibitors of miR such as α‐miR‐15a, α‐miR‐15b or α‐miR‐16 oligonucleotides (α‐Scr as Ctrl) for 48 h. (C, left) N‐Myc protein expression in SHEP (Dox‐inducible MYCN repression cell line) treated with Dox for 48 h. (C, right) The expression pattern of miR‐15a, miR‐15b, and miR‐16 in Dox‐treated SHEP cells. The names of the statistical tests used to determine significance or lack thereof (if applicable) are given. *P < 0.05.

We asked whether miR‐15a, miR‐15b, and miR‐16 modulate the expression of *MYCN* in NB cells. SK‐N‐B(E)2, NB‐19, and CHLA‐136 cells were transfected with precursors of miR or Scr Ctrl for 48 h. The qRT‐PCR and western blotting assays revealed that miR overexpression negatively regulated the expression of MYCN mRNA and N‐Myc protein (Fig. 2A). To confirm that the effect is not an off‐target, we transfected NB cells with LNA‐inhibitors against endogenous miR (anti‐Scr LNA‐inhibitors as Ctrl). Interestingly, upon transfection with anti‐miR, cells exhibited increased expression of MYCN mRNA and N‐Myc protein (Fig. 2B). The quantification of miR is provided in Fig. S2A,B. Further, we examined the MYCN mRNA stability in cells upon treatment with Act‐D in the presence of miR inhibitors. We found that anti‐miR significantly prolonged the half‐life of MYCN mRNA, suggesting MYCN regulation is at the messenger level (Fig. S2C). To confirm the relationship between *MYCN* and miR, we employed SHEP(Tet21/N), a Dox‐inducible N‐Myc repression NB cell line that constitutively expresses N‐Myc in the absence of Dox. Dox treatment (5 µg·mL^−1^) for 24 h significantly reduced N‐Myc levels in SHEP(Tet21/N) cells (Fig. 2C, left panel). Further, we observed higher levels of miR‐15a, miR‐15b, and miR‐16 in SHEP(Tet21/N) cells upon treatment with Dox (Fig. 2C, right panel). These observations suggest that miRs are inversely correlated with N‐Myc in NB cells.

### 3.3 MiR‐15a, miR‐15b, and miR‐16 interact with MYCN mRNA

**Figure 3:**
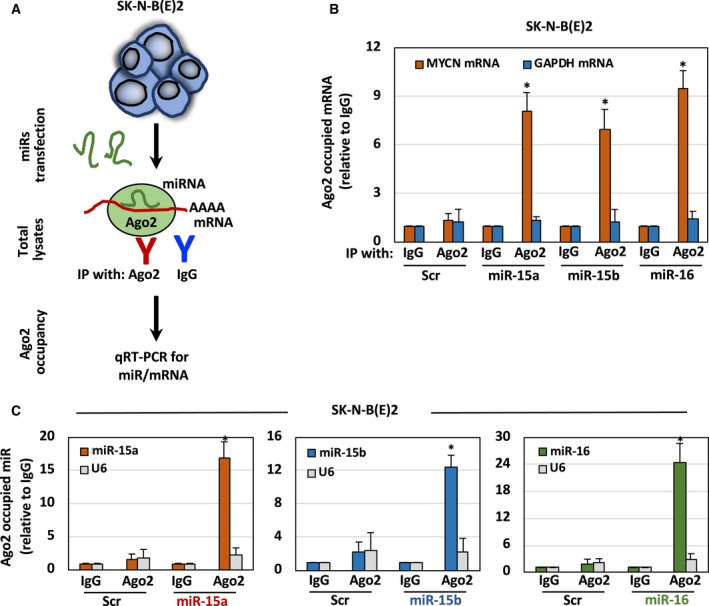
MiR‐15a, miR‐15b, and miR‐16 interact with MYCN mRNA. (A) Schematic representation of the Ago2 IP experiment for the identification of Ago2‐occupied mRNA or miR. (B,C) Overexpression of miR increases Ago2 interaction with MYCN mRNA and miR. SK‐N‐B(E)2 cells were transfected with precursors of miR for 48 h followed by IP with α‐Ago2 antibody. A qRT‐PCR assay for Ago2‐occupied (B) MYCN mRNA or (C) miR. The data were compared with the Ctrl IgG‐bound mRNA or miR set to 1 for normalization. Data are presented as mean ± SD of triplicates. *P < 0.05. The names of the statistical tests used to determine significance or lack thereof (if applicable) are given.

Ago2 is the core component of the RNA‐induced silencing complex (RISC) that plays a significant role in miR‐or siRNA‐mediated gene regulation by bringing miR to the complementary sequence of the target mRNA (Chendrimada *et al.*, 2005; Gregory *et al.*, 2005). Therefore, Ago2 interaction with miR or target mRNA serves as an indicator of target gene regulation by a specific miR. We asked whether miR can bind with MYCN mRNA. To test this, we transfected SK‐N‐B(E)2 cells with miR‐15a, miR‐15b, and miR‐16 oligonucleotides for 48 h and performed RNA‐IP using Ago2 antibodies. IgG RNA‐IP was also performed as a Ctrl. The scheme of Ago2‐RNA IP is given in Fig. 3A. A corresponding IP‐western blotting picture of Ago2 IP is provided in Fig. S3. As shown in Fig. 3B, enrichment of *MYCN* mRNA, but not *GAPDH* mRNA, was found in the anti‐Ago2 IP. Similarly, *MYCN* mRNA was not enriched in the IgG Ctrl IP fractions. Figure 3C also indicates that Ago2 interacts with miR but not with U6 in Ago2 IP. Further, miRs were not enriched in the IgG Ctrl IP fractions. These results indicate that miR‐15a, miR‐15b, and miR‐16 regulate N‐Myc protein by interaction with MYCN mRNA.

### 3.4 MiR‐15a, miR‐15b, and miR‐16 regulate *MYCN* through 3′UTR

**Figure 4:**
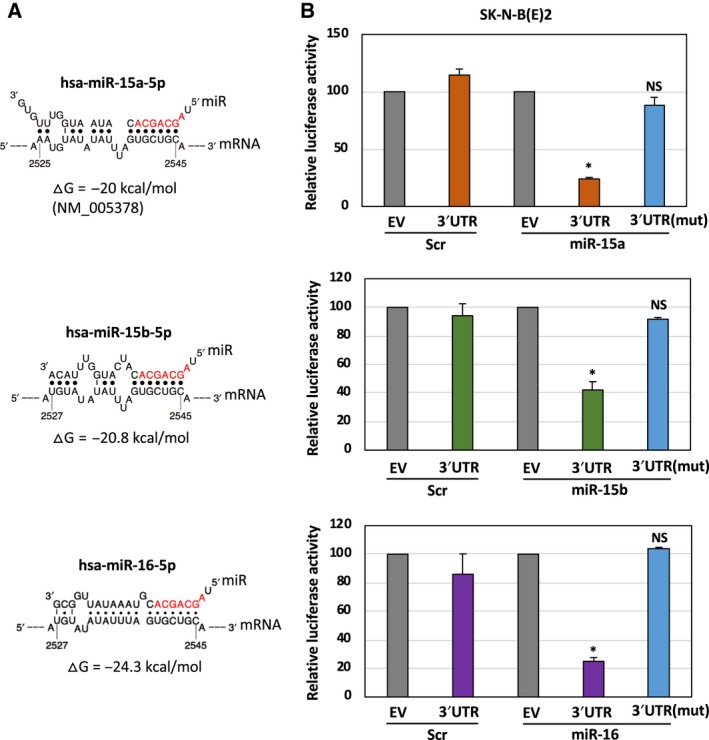
MiR‐15a, miR‐15b, and miR‐16 destabilize MYCN mRNA through 3'UTR binding. (A) The sequence alignment and the predicted binding sites between miR‐15a, miR‐15b, and miR‐16 and 3'UTR of MYCN mRNA. MiR sequences recognized by MYCN mRNA are shown in red. (B) Cells were transfected with either luciferase reporter empty or vector containing the MYCN‐3′UTR wild‐type or mutant in the target sites in the presence or absence of precursors of miR for 48 h, followed by measuring the luciferase activity. The data were compared with the EV, which has been set to 100 for normalization. Data are presented as mean ± SD of triplicates. *P < 0.05. The names of the statistical tests used to determine significance or lack thereof (if applicable) are given.

Using TargetScan, an *in silico* analysis tool, we predicted the sequence‐specific interaction between *MYCN* 3′UTR (NCBI NM_005378) and miR‐15a, miR‐15b, and miR‐16. The predicted sequences are given in Fig. 4A. We investigated whether miR regulates *MYCN* through 3’UTR. *MYCN*‐3’UTR (NM_005378) cloned in pMirTarget luciferase vector is commercially available from OriGene. pMirTarget luciferase empty vector served as a Ctrl in these experiments. A schematic model of the empty luciferase vector or containing *MYCN*‐3’UTR is given in Fig. S4. SK‐N‐B(E)2 cells were transfected with precursors of miR‐15a, miR‐15b, and miR‐16 oligonucleotides (25 nm) together with either pmiR‐EV or pmiR‐*MYCN*‐3’UTR for 48 h, and luciferase activities were assayed using the luciferase assay system (Promega). As shown in Fig. 4B, cells transfected with miR showed a reduction in luciferase activity with pmiR‐*MYCN*‐3’UTR but not with pmiR‐EV. These observations indicate that miR‐15a, miR‐15b, and miR‐16 target *MYCN* through 3’UTR.

### 3.5 MiR‐15a, miR‐15b, and miR‐16 inhibit cell proliferation, migration and colony formation in NB cells

**Figure 5:**
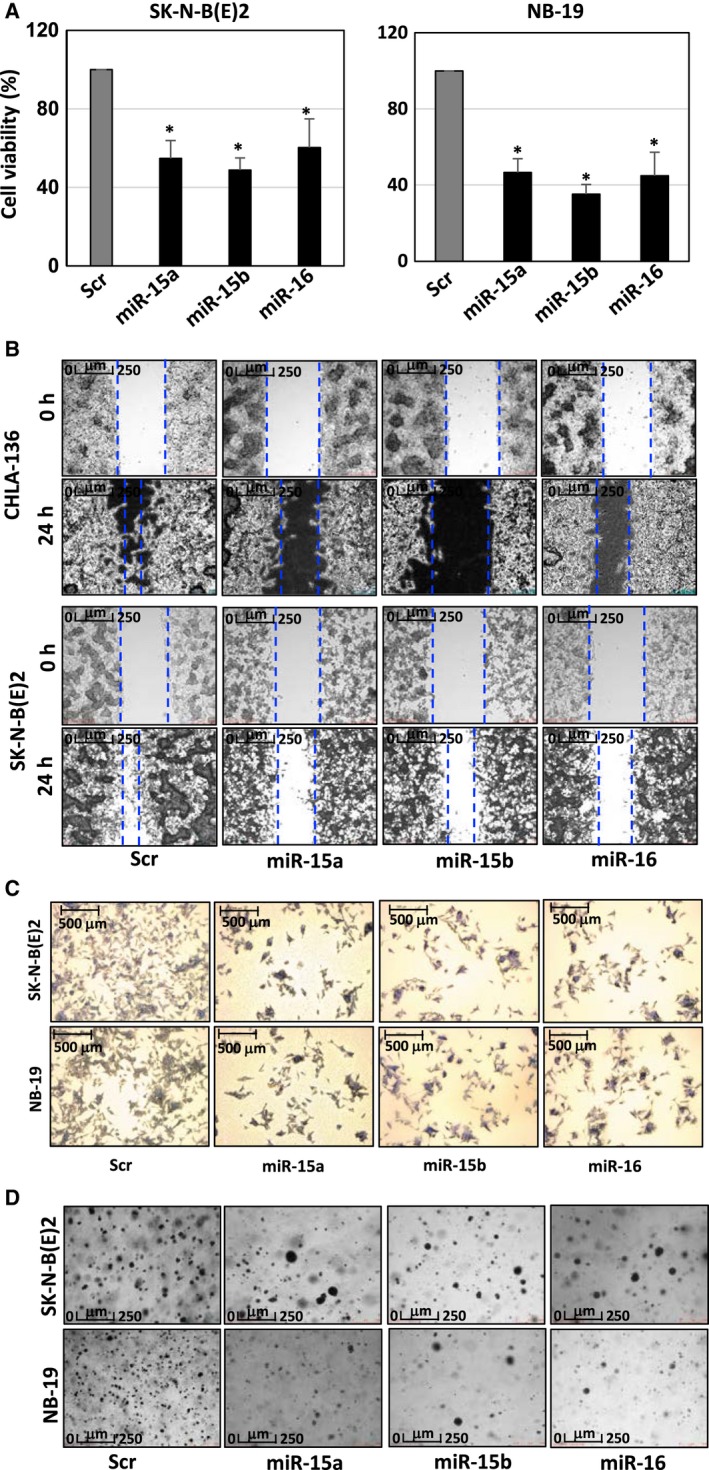
MiR‐15a, miR‐15b, and miR‐16 inhibit cell proliferation, migration, invasion potential, and colony formation in NB cells. A representative quantification graph of (A) MTT assay evaluating the effects of miR on cell proliferation; (B) images of wound healing in CHLA‐136 and SK‐N‐B(E)2 cells after transfection with Scr Ctrl or miR‐15a, miR‐15b, and miR‐16 mimics for 48 h. Scale: 250 µm. (C) Invasion assay evaluating the effects of miR on the migration and invasion potential of NB cells, Scale: 500 µm. (D) Soft agar colony formation assay evaluating the anchorage‐independent growth in NB cells (SK‐N‐B(E)2 and NB‐19) after 48 h transfection with Scr Ctrl or miR‐15a, miR‐15b, and miR‐16 mimics. Scale: 250 µm. The miRNA‐transfected cells showed a significant reduction in the numbers as compared with the Scr Ctrl groups. Data presented are the mean ± SD of three independent experiments. *P < 0.05. The names of the statistical tests used to determine significance or lack thereof (if applicable) are given.

Next, we investigated the effects of miR‐15a, miR‐15b and miR‐16 in the development and progression of NB. The miRs were overexpressed in SK‐N‐BE(2), NB‐19, and CHLA‐136 cells. The proliferation, migration, and colony formation of overexpressing cells was examined. Using MTT assay, we observed a significant reduction in the proliferation of miR‐overexpressing cells as compared with Scr control cells (Figs 5A and S5A). Further, wound healing assays showed that miR‐15a, miR‐15b, and miR‐16 reduced the migration ability of NB cells compared with their respective Scr Ctrl (Fig. 5B). Consistent with these results, transwell migration assays showed a significant decrease in the number of migrated cells in the miR received NB cells compared with the Scr Control cells (Figs 5C and S5B). These results indicate that miRs inhibit cell migration. Furthermore, colony formation assay demonstrated a decrease of colony numbers in SK‐N‐BE(2) and NB‐19 cells upon receiving miR (Figs 5D and S5C). These results indicate that ectopic expression of miR significantly reduces the anchorage‐independent growth in NB cells. Taken together, miR‐15a, miR‐15b, and miR‐16 act as tumor suppressors by negatively regulating proliferation, migration, and colony formation in NB cells*.*

### 3.6 MiR‐15a, miR‐15b, and miR‐16 inhibit tumor growth in NB xenografts *in vivo*

**Figure 6:**
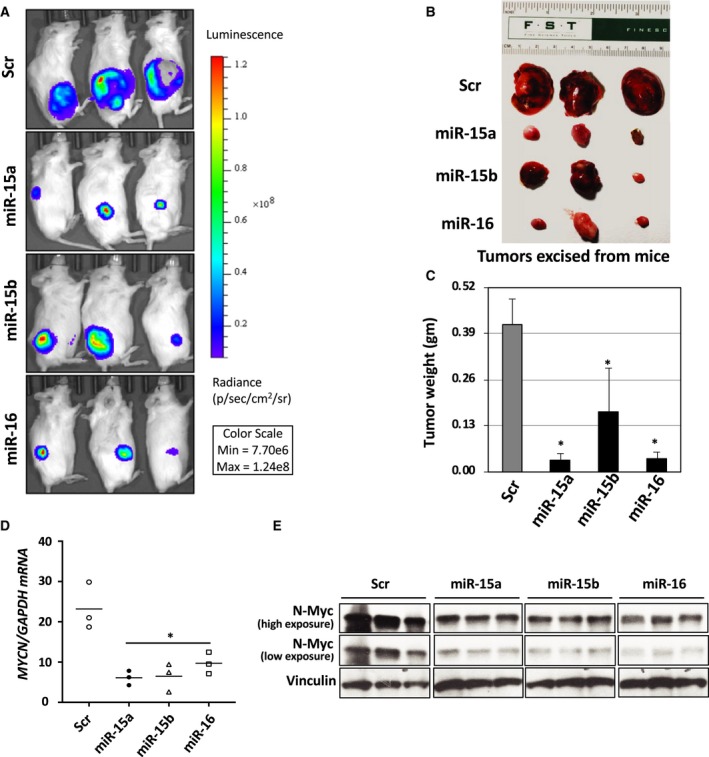
MiR‐15a, miR‐15b, and miR‐16 reduce tumor burden in a mouse xenograft model of NB. (A) Representative live bioluminescent images of tumor burden in NOD/SCID mice received a subcutaneous injection of luciferase‐expressing SK‐N‐B(E)2 cells transfected with either Scr Ctrl or precursors of miR‐15a, miR‐15b or miR‐16 oligonucleotides. (B) Images of the excised tumors and (C) tumor weights of the mice from various treatment groups are shown. Tumor tissue samples were extracted from mice. A portion was used for RNA isolation, and the other was used by western blotting assay for the N‐Myc protein. A qRT‐PCR for (D) MYCN mRNA (normalized to *GAPDH* mRNA), and (E) N‐Myc protein expression in excised tumors from xenografts. *P < 0.05. The names of the statistical tests used to determine significance or lack thereof (if applicable) are given.

Our *in vitro* data showed that miR‐15a, miR‐15b, and miR‐16 act as tumor suppressors by targeting *MYCN*. To confirm the tumor‐suppressive role of these miRs in NB cells *in vivo,* we established a subcutaneous tumor xenograft model using NSG mice. All the animal experiments were performed using a protocol approved by the IACUC of UNMC. First, we transfected luciferase‐expressing SK‐N‐B(E)2 stable cells with miR‐15a, miR‐15b, miR‐16 or Scr Ctrl for 24 h. Cells were implanted subcutaneously (4 × 10^6^ per 100 μL PBS/Matrigel) into the flank of the mice for 45 days using our previously described protocol (Challagundla *et al.*, 2015). A schematic model of the experimental plan is given in Fig. S6A. Tumor growth of the luciferase‐expressing cells was noted using a bioluminescence imaging system (Xenogen IVIS 100 System; Caliper Life Sciences, Inc., Waltham, MA, USA). A significant reduction in bioluminescence (Fig. 6A), tumor size (Fig. 6B), and tumor weight (Fig. 6C) was observed in mice groups that received miR transfected cells as compared with the Scr Ctrl group. The miR expression in tumors was confirmed by qRT‐PCR (Fig. S6B). A qRT‐PCR and western blotting data from Figs 6D,E and S6C revealed that MYCN mRNA and N‐Myc protein levels were significantly reduced in miR groups compared with the Scr Ctrl group. Overall, these data indicate that the changes in the tumor formation are due to the direct effect of miR‐15a, miR‐15b, and miR‐16 on MYCN, and therefore could be considered tumor suppressors in NB.

## 4 Discussion

MYC family members are deregulated in many human cancers (Dang, 2012). Among the MYC family, *MYCN* is critical in NB progression and therapy resistance, and its higher expression is linked with poor prognosis and survival irrespective of the risk group in NB patients (Brodeur *et al.*, 1984; Fredlund *et al.*, 2008). Higher *MYCN* expression drives the tumor initiation and progression in TH‐MYCN mouse and zebrafish models, suggesting that MYCN is conserved across the species (Weiss *et al.*, 1997; Zhu *et al.*, 2012). *MYCN* in‐activation using anti‐sense RNA molecules led to the regression of tumor formation in an animal model (Burkhart *et al.*, 2003). A majority of high‐risk NB (50%) is associated with the amplification of *MYCN* and the development of metastatic potential (Schwab *et al.*, 1984; Seeger *et al.*, 1985). *MYCN*‐amplified tumors express a specific set of gene signatures that are involved in the disease progression and are associated with poor survival in NB patients (Westermann *et al.*, 2008). Moreover, treating NB cells with differentiating agent retinoic acid induces the degradation of *MYCN* (Thiele *et al.*, 1988). Additionally, an RNA‐binding protein Lin28B inhibits the expression of let‐7 miRNA through up‐regulation of MYCN mRNA in NB cells with *MYCN* amplification (Powers *et al.*, 2016). Recent evidence suggests that MYCN mRNA acts as an oncogene by sponging endogenous let‐7 miRNA, which is an independent function from N‐Myc protein (Viswanathan *et al.*, 2008).

A study by Beckers *et al.* (2015) explored the *MYCN* targeting/interacting miR using an unbiased screening approach, and identified 12 MYCN targeting miR. Authors have also provided evidence of the down‐regulation of these miRs in a mouse model of NB driven by TH‐MYCN, suggesting that *MYCN* negatively regulates these miR (Beckers *et al.*, 2015). A group led by Carlo Dominici has applied high‐throughput next‐generation sequencing to identify the miR transcriptome in a cohort of *MYCN*‐amplified and non‐amplified NB tumors (Megiorni *et al.*, 2017). Authors found 47 miRs with higher expression and 81 miRs with lower expression in the NB tumors irrespective of *MYCN* amplification. An up‐regulation of 17 and a down‐regulation of six miRs found in the *MYCN*‐amplified NB subtypes suggested that these differential expressions of miRs are involved in various pathways such as DNA repair and mTOR pathways (Megiorni *et al.*, 2017; Schramm *et al.*, 2013). Several other groups have demonstrated that cells treated with all‐*trans*‐retinoic acid, a retinoid derivative of a chemotherapy drug, express a specific miR signature and reduced levels of *MYCN* in NB cells (Chen and Stallings, 2007; Laneve *et al.*, 2007; Reynolds and Lemons, 2001; Thiele *et al.*, 1985; Truckenmiller *et al.*, 2001). A group led by C. Einvik tested a distinct set of miRNA that potentially target *MYCN* 3’UTR (Buechner *et al.*, 2011). Authors found that 21 miRNAs from six miRNA families, i.e. let‐7/98/202, miRNA‐17/20/93/106/519, miRNA‐19a/b, miRNA‐29a/b/c, miRNA‐34a/b/c/449a/b/c/699, and miRNA‐101, reduced *MYCN*‐3’UTR luciferase activity in cells upon transfection with respective miRNA (Buechner *et al.*, 2011). However, let‐7e, miRNA‐19a, miRNA‐19b, miRNA‐29a, miRNA‐29b, miRNA‐29c, miRNA‐101, and miRNA‐202 were able to reduce the N‐Myc protein levels in Kelly cells, an *MYCN*‐amplified NB cell line. Interestingly, let‐7e and miRNA‐202 exhibited anti‐proliferative effects in Kelly cells (Buechner *et al.*, 2011). Another study identified a set of 29 miRNA in targeting *MYCN*‐3’UTR using a high‐throughput luciferase reporter screen. Further, the authors demonstrated that 12 of 29 miRs were inversely correlated with MYCN expression in a transgenic *MYCN* NB mouse model (Beckers *et al.*, 2015).

We observed that miR‐15a, miR‐15b, and miR‐16 regulate *MYCN* in NB‐specific PDX. Further, these miRs are negatively correlated with *MYCN* expression in a PDX model (Fig. 1B). However, the mechanisms of *MYCN* repression by these miRs are not fully understood. The inverse correlation between *MYCN* and *DLEU2*, the host gene for miR‐15 family, in NB patient tumor samples clearly demonstrate that *MYCN* regulation is through these miRs. Whereas *MYCN* expression was reduced by miR oligos, an up‐regulation in expression was observed with the miR inhibitors. That N‐Myc is almost completely abolished whereas miR expression is significantly induced in SHEP(Tet21/N) cells upon doxycycline treatment suggest that these miRs specifically target MYCN mRNA in NB cells. The three cell lines used in the current study [SK‐N‐B(E)2, NB‐19, CHLA‐136] are *MYCN*‐amplified. The *MYCN* amplification may also contribute to the elevation in *MYCN* expression. However, had this been solely because of *MYCN* amplification, the miR overexpression would not suppress *MYCN* expression in cell lines (Fig. 2A). That selective inhibition of miR elevated *MYCN* expression, further supports the role of miR in *MYCN* regulation (Fig. 2B). Moreover, overexpression of miR suppressed the tumorigenic potential of *MYCN* amplified cell lines (Fig. 5). Overall, it is plausible that miR regulate *MYCN* expression in NB cells. We also tested the regulation of endogenous MYCN mRNA by treating cells with a low dose of Act‐D with or without miR inhibitors (Fig. S2C). We show that anti‐miR significantly prolongs the half‐life of MYCN mRNA, suggesting that *MYCN* is regulated at the mRNA level.

In our recent study, we showed that NB‐secreted miR‐21 in exosomes induces chemotherapy resistance through another oncogenic miR‐155 within the tumor microenvironment by targeting TERF1, an inhibitor of the telomerase axis (Challagundla *et al.*, 2015). Another study showed a different pathway of interaction with the tumor microenvironment, secretion of oncogenic miRNA in exosome‐like particles (Haug *et al.*, 2015). One question to be answered is how these miRs specifically associate with MYCN mRNA. Our luciferase assay results support that these miRs regulate MYCN mRNA through the 3’UTR region (Fig. 3B). Ago2 is an important component of the RISC and plays a key role in miR‐mediated gene silencing by direct interaction with mature miR and target mRNA (Roberts, 2015; Yi, 2018). Ago2 interacts with MYCN mRNA and miR in cells (Fig. 3B,C). Thus, it is likely that binding to *MYCN* mRNA is specific. However, whether this interaction requires any additional cofactor proteins, is still an unanswered question. We plan to identify other factors involved in this regulation in the near future. In our study, we demonstrate that miR‐15a, miR‐15b, and miR‐16 decreased cell proliferation, cell migration, and cell invasion in NB cells. We believe that this phenotype is due to degradation of *MYCN*. The cell viability was examined after 48 h transfection of the cells with miRNA. The live cells left after 48 h transfection in each group were used for invasion assay. This completely rules out the possibility that the invasion potential of transfected cells was reduced due to the reduced number of alive cells.

N‐Myc transcriptionally activates the oncogenic *miR‐17*∼*92* cluster, which induces tumorigenicity through estrogen receptor‐α (Loven *et al.*, 2010). Indeed, higher ESR1 expression is required for better survival and induces cell cycle arrest and differentiation upon treatment with estradiol (Loven *et al.*, 2010; Ma *et al.*, 1993). MYCN also regulates the expression of a tumor suppressor, Dickkopf‐3 (DKK3), a secreted glycoprotein in culture supernatants, by transcriptionally activating the expression of *mir‐92* in NB cells (Haug *et al.*, 2011). Further, DKK3 is inversely correlated with MYCN expression in NB patients (Koppen *et al.*, 2008). Therefore, *MYCN* has become a potential therapeutic target in NB. In our study, we treated mice with miR‐overexpressing NB cells to see whether these miRs target *MYCN*. Interestingly we found a reduction in tumor weight, tumor size, and tumor shrinkage (Fig. 6). We also demonstrate that MYCN mRNA and N‐Myc proteins are significantly reduced in the tumor of the mice bearing miR‐overexpressing cells (Fig. 6D,E), suggesting the tumor suppressor properties of these miRs. This may explain why MYCN is strongly expressed in a majority of high‐risk NB patients.

## 5 Conclusion

Our working model is summarized in the graphic abstract. In summary, our studies provide an additional mechanism of the MYCN regulation by miR. We found lower levels of miR‐15a, miR‐15b, and miR‐16, along with higher MYCN expression in NB PDX. By using both *in vitro* and *in vivo* models, we demonstrate that these miRs inhibit proliferation, cell migration, colony formation, and tumorigenesis by directly targeting MYCN through its 3’UTR. Down‐regulation of these miRs may result in the stabilization of MYCN. This pathway expands our understanding of the regulation of MYCN at the level of transcription. In conclusion, our data demonstrated that miR‐15a, miR‐15b, and miR‐16 act as tumor suppressors by regulating the MYCN pathway. Thus, targeting these miRs and MYCN provides a novel therapeutic avenue to combat aggressive NB.

## Conflict of interest

The authors declare no conflict of interest.

## Author contributions

KBC and SC designed studies, performed *in vitro* and *in vivo* experiments, analyzed data, wrote the manuscript, and coordinated with all coauthors. PCR provided NB PDX tumor tissues. SG and LYP provided animals and helped with *in vivo* experiments. AP, DWC, SCG, and MKP assisted in manuscript preparation, review, edits, and revision.

## Supporting information


**Fig. S1.** (A) A schematic model showing the overall procedure for the expansion of PDX tumors in nude mice. Tumor tissues were surgically removed from the patient followed by *in vivo* subcutaneous implantation. Tumor tissues were harvested after reaching ~ 1.5 cm followed by re‐implantation in the next set of mice in multiple passages for expansion, and were used for the experiments. (B) Kaplan–Meier curves showing the survival of NB patients (*n* = 88) with high MYCN mRNA expression in tumors. Patients with higher *MYCN* had shorter survival.
**Fig. S2.** (A,B) A qRT‐PCR for miR‐15a, miR‐15b, and miR‐16 in NB cell lines [SK‐N‐BE(2), NB‐19 and CHLA‐136] transfected with either (A) miR‐15a, miR‐15b or miR‐16 oligonucleotides (Scr as control) or (B) inhibitors of miR such as α‐miR‐15a, α‐miR‐15b or α‐miR‐16 oligonucleotides (α‐Scr as control) for 48 h. (C) Inhibition of miR‐15a, miR‐15b, and miR‐16 stabilizes MYCN mRNA. A representative qRT‐PCR graph of MYCN mRNA decay (normalized to GAPDH mRNA) in SK‐N‐B(E)2 cells transfected with anti‐miRNA (anti‐Scr as control) followed by treatment with 2 μm Act‐D for the indicated time points. *P < 0.05. The names of the statistical tests used to determine significance or lack thereof (if applicable) are given.
**Fig. S3.** IP‐western blotting of Ago2 in SK‐N‐B(E)2 cells, transfected with precursors of miR‐15a, miR‐15b, and miR‐16 for 48 h followed first by IP and then by western blotting with α‐Ago2 antibody.
**Fig. S4.** A representative map of the pGL3‐EV or pGL3‐*MYCN*‐3'UTR luciferase vectors used for the luciferase reporter assays.
**Fig. S5.** (A) A quantification graph of MTT assay evaluating the cell proliferation in CHLA‐136 cells. (B,C) A representative quantification graph of (B) cell migration and (C) colony formation assay in NB cells after transfection with Scr control or miR‐15a, miR‐15b, and miR‐16 mimics for 48 h. The quantification numbers in Scr group were set at 100, and the values were reported as percentage change. Data presented are mean + SD of triplicates. *P < 0.05. The names of the statistical tests used to determine significance or lack thereof (if applicable) are given.
**Fig. S6.** (A) Timeline for NB xenograft experiments. (B) A qRT‐PCR for miR‐15a, miR‐15b, and miR‐16 (normalized to snoRNA U6) expression in tumors excised from NB xenografts received a subcutaneous injection of luciferase‐expressing SK‐N‐B(E)2 cells transfected with either Scr control or precursors of miR‐15a, miR‐15b or miR‐16 for 60 days. (C) Densitometry analysis of N‐Myc protein expression. *P < 0.05. The names of the statistical tests used to determine significance or lack thereof (if applicable) are given.
