# Supplementary material for: miR‐15a‐5p, miR‐15b‐5p, and miR‐16‐5p inhibit tumor progression by directly targeting MYCN in neuroblastoma
Source: Mol Oncol. 2019 Nov 29;14(1):180–96. doi: 10.1002/1878-0261.12588 (PMC6944109; doi:10.1002/1878-0261.12588)

Supplementary Figure 1

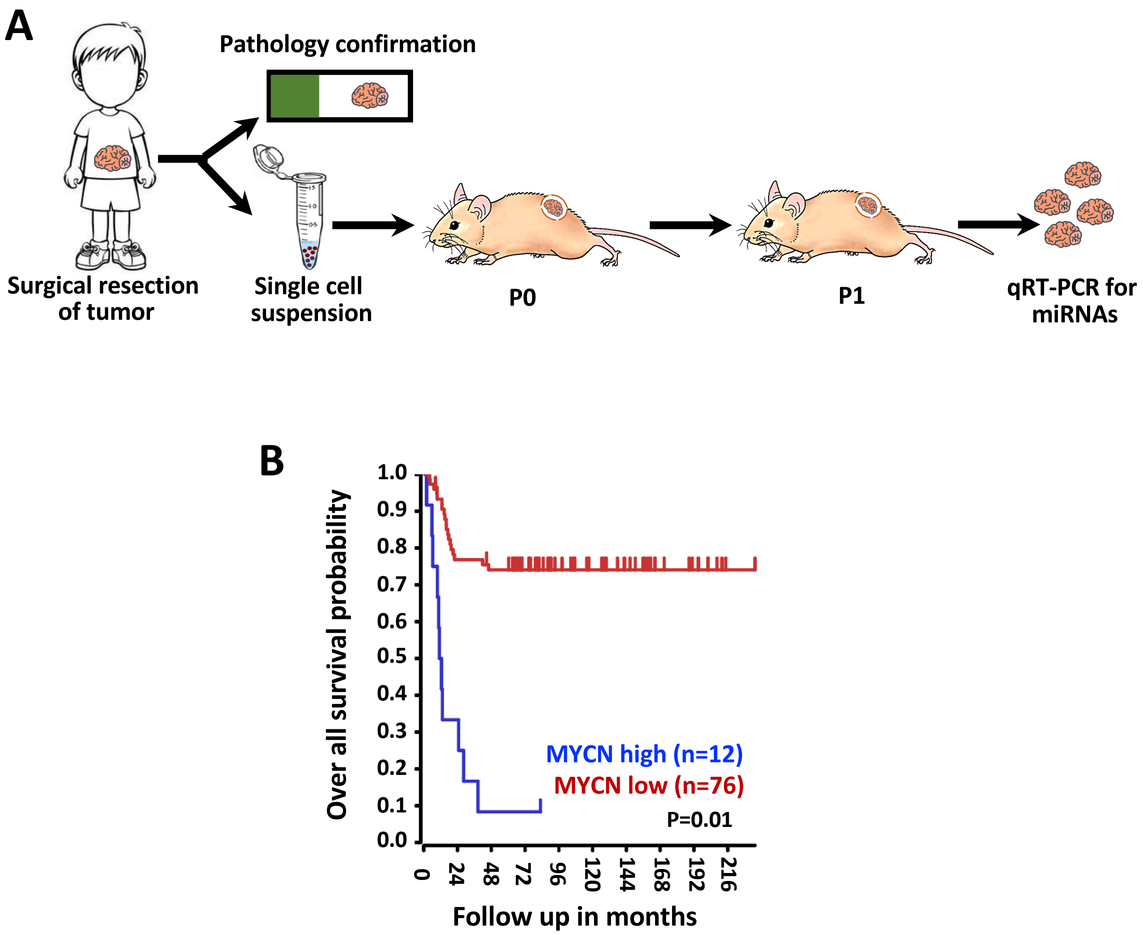

Supplementary Figure 2

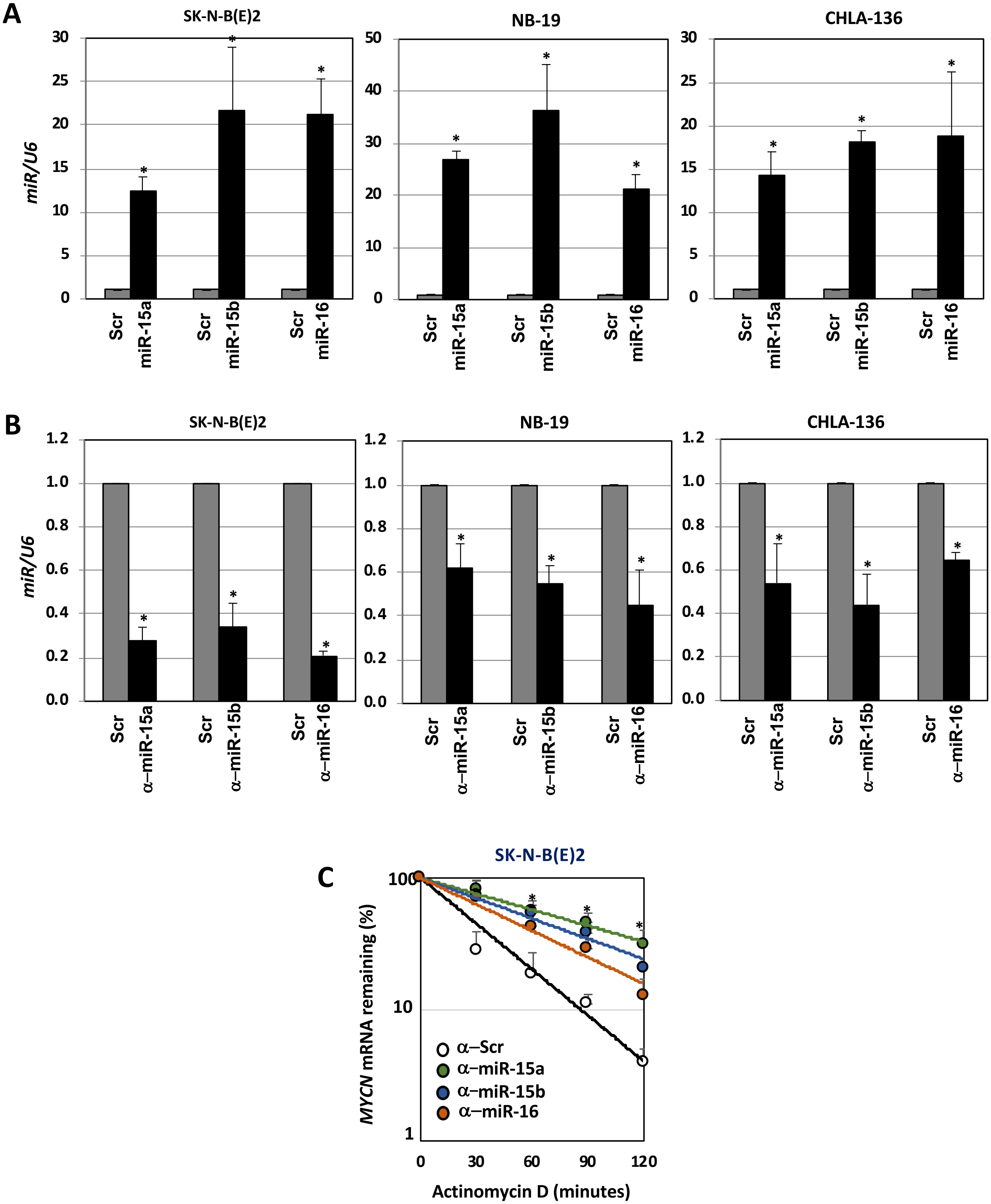

Supplementary Figure 3

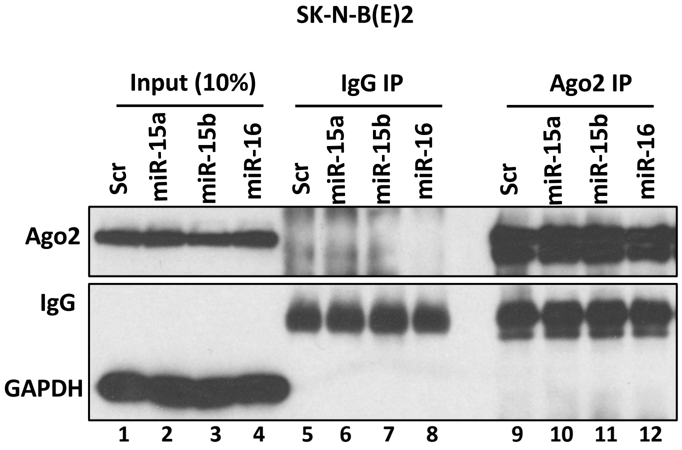

Supplementary Figure 4

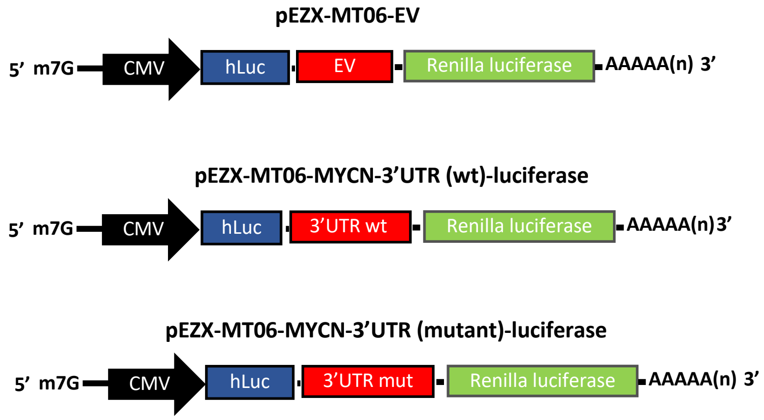

Supplementary Figure 5

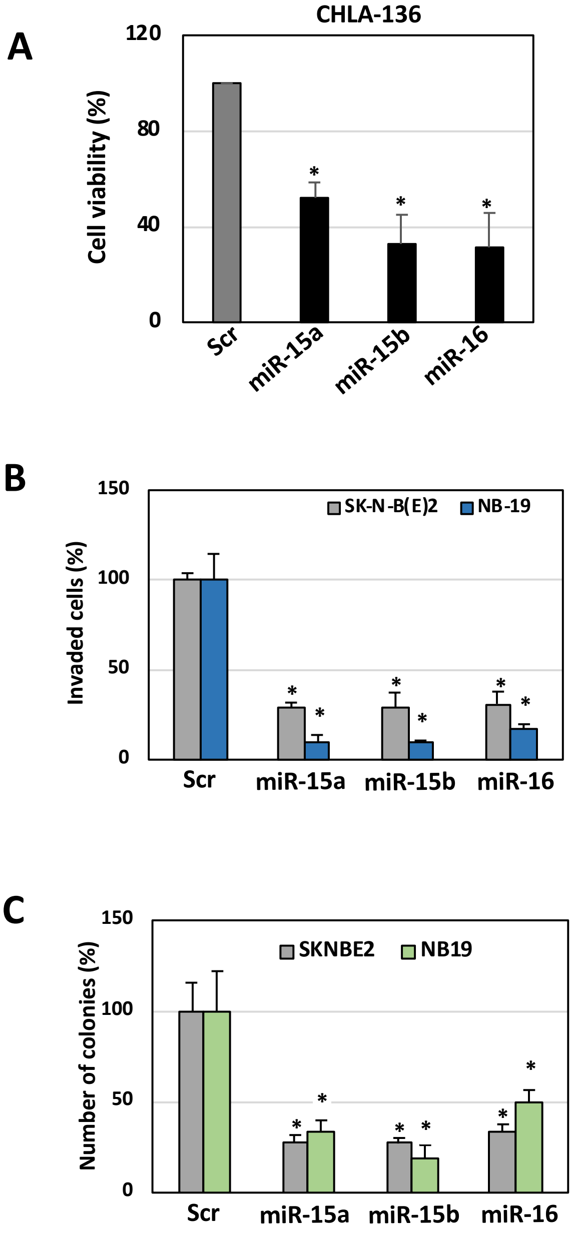

Supplementary Figure 6

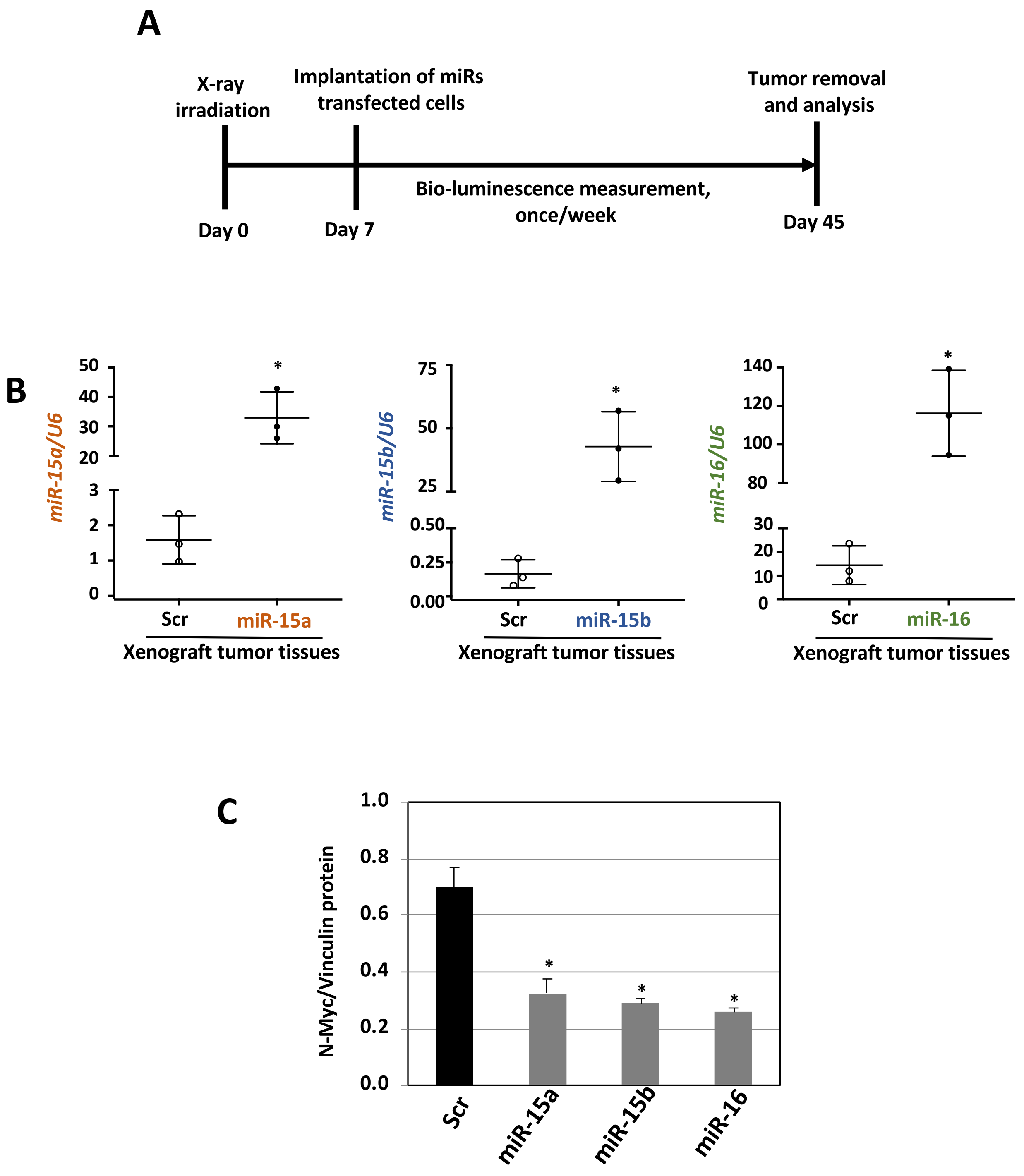

Supplement: Supplementary file 1 — Fig. S1. (A) A schematic model showing the overall procedure for the expansion of PDX tumors in nude mice. Tumor tissues were surgically removed from the patient followed by in vivo subcutaneous implantation. Tumor tissues were harvested after reaching ~ 1.5 cm followed by re‐implantation in the next set of mice in multiple passages for expansion, and were used for the experiments. (B) Kaplan–Meier curves showing the survival of NB patients (n = 88) with high MYCN mRNA expression in tumors. Patients with higher MYCN had shorter survival. Fig. S2. (A,B) A qRT‐PCR for miR‐15a, miR‐15b, and miR‐16 in NB cell lines [SK‐N‐BE(2), NB‐19 and CHLA‐136] transfected with either (A) miR‐15a, miR‐15b or miR‐16 oligonucleotides (Scr as control) or (B) inhibitors of miR such as α‐miR‐15a, α‐miR‐15b or α‐miR‐16 oligonucleotides (α‐Scr as control) for 48 h. (C) Inhibition of miR‐15a, miR‐15b, and miR‐16 stabilizes MYCN mRNA. A representative qRT‐PCR graph of MYCN mRNA decay (normalized to GAPDH mRNA) in SK‐N‐B(E)2 cells transfected with anti‐miRNA (anti‐Scr as control) followed by treatment with 2 μm Act‐D for the indicated time points. *P < 0.05. The names of the statistical tests used to determine significance or lack thereof (if applicable) are given. Fig. S3. IP‐western blotting of Ago2 in SK‐N‐B(E)2 cells, transfected with precursors of miR‐15a, miR‐15b, and miR‐16 for 48 h followed first by IP and then by western blotting with α‐Ago2 antibody. Fig. S4. A representative map of the pGL3‐EV or pGL3‐MYCN‐3'UTR luciferase vectors used for the luciferase reporter assays. Fig. S5. (A) A quantification graph of MTT assay evaluating the cell proliferation in CHLA‐136 cells. (B,C) A representative quantification graph of (B) cell migration and (C) colony formation assay in NB cells after transfection with Scr control or miR‐15a, miR‐15b, and miR‐16 mimics for 48 h. The quantification numbers in Scr group were set at 100, and the values were reported as percentage change. Data presented [file MOL2-14-180-s001.pdf]
